# Use of a microsurgical vascular clip system (Yasargil clip) in laparoscopic fibroid enucleation

**DOI:** 10.4274/jtgga.galenos.2020.2020.0046

**Published:** 2020-12-04

**Authors:** Shadi Younes, Julia Caroline Radosa, Anke Mothes, Bahriye Aktas, Marc Philipp Radosa

**Affiliations:** 1 Department of Obstetrics and Gynecology, Leipzig University Hospital, Leipzig, Germany; 2 Department of Obstetrics and Gynecology, Saarland University Hospital, Homburg, Germany; 3 Department of Obstetrics and Gynecology, St. George’s University Hospital, Eisenach, Germany

**Keywords:** Yasargil clips, fibroid enculeation, laparoscopy

## Abstract

This video demonstrates the use of a microsurgical temporary vascular clip system to facilitate laparoscopic enucleation of uterine fibroids. Throughout the course of the last three decades, the laparoscopic route has been established as the approach of choice in the surgical treatment of uterine fibroids. Laparoscopic fibroid enucleation is characterized by a low morbidity rate and a high patient satisfaction level. Especially when treating a large fibroid or multiple fibroids, the well-vascularized myometrium can constitute a technical challenge in endoscopic fibroid enucleation. Diffuse bleeding may lead to significant intraoperative hemorrhage. The extensive use of bipolar or monopolar diathermy, in order to achieve hemostasis, might lead to post-operative uterine wall necrosis with a potential risk of uterine rupture during subsequent pregnancies. To address this clinical challenge, we developed a technique with temporary interruption of the uterine blood supply by applying a microsurgical vascular clip (Yasargil vascular clip system, Aesculap, Tuttlingen, Germany) to the uterine artery and the utero-ovarian vessel arcade to minimize bleeding during endoscopic fibroid enucleation.

## Introduction

The purpose of this video was to demonstrate the use of a microsurgical temporary vascular clip system to facilitate laparoscopic enucleation of uterine fibroids (Video 1).

Throughout the course of the last three decades, the laparoscopic route has been established as the approach of choice in the surgical treatment of uterine fibroids. Laparoscopic fibroid enucleation is characterized by a low morbidity rate and a high patient satisfaction level (1). Especially when treating a large fibroid, the well-vascularized myometrium can constitute a technical challenge in endoscopic fibroid enucleation. Diffuse bleeding may lead to significant intraoperative hemorrhage. The extensive use of bipolar or monopolar diathermy, in order to achieve hemostasis, might lead to post-operative uterine wall necrosis with a potential risk of uterine rupture during subsequent pregnancies (2).

To address this clinical challenge, we developed a technique with temporary interruption of the uterine blood supply by applying a microsurgical vascular clip (Yasargil vascular clip system, Aesculap, Tuttlingen, Germany) to the uterine artery and the utero-ovarian vessel arcade to minimize bleeding during endoscopic fibroid enucleation. Yasargil vascular clips were originally used in the treatment of intra-cranial aneurysms (3). In surgical gynecology, the Yasargil clip has been introduced for the treatment of vascular injuries during laparoscopic procedures (4).

**Figure 1:**
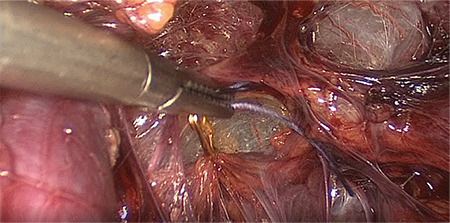
The left uterine artery and the left ureter are identified by blunt dissection and a Yasargil clip is applied to the uterine artery

**Figure 2:**
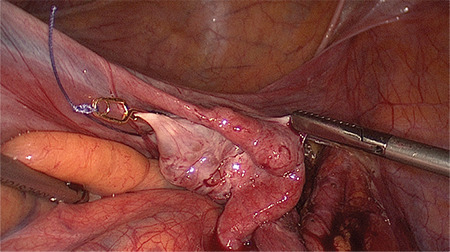
Additional uterine blood supply via the uteroovarian vessel arcade is occluded by placing a Yasargil clip on the ovarian ligament of each side

**Figure 3:**
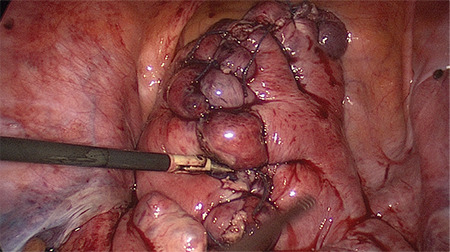
Reconstruction of the uterine wall: a first stich is used to close the deep uterine muscle layer, while the following back-stich is used to close the superficial uterine muscle layer and the uterine serosa

The procedure was carried out under general anesthesia and the patient was placed in a dorsal lithotomy position. A Verres needle was introduced subumbilically and the abdomen was inflated with carbon dioxide to a pressure of 8 mmHg. We used low-pressure laparoscopy in order to minimize post-operative pain (5). Upon installation of the pneumoperitoneum, a 12 mm trocar was inserted subumbilically for the video laparoscope (0 degree Endoeye, Olympus, Shinjuku, Japan) and three 5 mm ports were inserted in the left, middle and right lower abdominal quadrant, respectively. The peritoneal cavity was inspected and the uterine fibroid was identified. Then, the peritoneum on the left pelvic brim was incised laterally to the external iliac artery and medially to the ligamentum ovarii suspensorium to access the retroperitoneum. The left uterine artery and the left ureter were identified by blunt dissection and a temporary 15 mm Yasargil clip (Yasargil clip system, FT 280T; Aesculap, Tuttlingen, Germany) with a clamp force of 0.88 Newton was applied to the uterine artery, lateral to the ureter (Figure 1). This step was repeated on the contralateral site. The additional uterine blood supply, via the utero-ovarian vessel arcade, was occluded by placing a Yasargil clip on the ovarian ligament on each side (Figure 2). All four Yasargil clips are marked with a vicryl suture to facilitate identification towards the end of the surgery. The uterine serosa and the myometrium were subsequently incised and the surface of the fibroid was exposed. The lower middle 5 mm port was replaced by a 10 mm port and the fibroid was grasped with 10 mm tenaculum forceps. Then the fibroid was extracted from surrounding myometrium by blunt dissection. Closure of the uterus was achieved by interrupted, intra-corporeal double-layer sutures. A first stich was used to close the deep uterine muscle layer, while the following back-stich was used to close the superficial uterine muscle layer and the uterine serosa (Figure 3). Following the closure of the uterus, the Yasargil clips were removed, both peritoneal incisions were closed by continuous suture and the blood circulation of the uterus was photo documented. Fibroids were morcellated, using an electric morcellator (Storz, Tuttlingen, Germany) and extracted through the midline trocar and at the end of the procedure an intra-abdominal drain (French gauge 18) was placed for postoperative monitoring purposes.

Written informed consent was obtained from the patient for publication of this video and any accompanying images.

***Video 1. https://www.doi.org/10.4274/jtgga.galenos.2020.2020.0046.video1***
